# Chlorine Dioxide (ClO_2_)-Releasing Sachet for Preservation of Cherry Tomatoes

**DOI:** 10.3390/molecules30092041

**Published:** 2025-05-03

**Authors:** Junseok Lee, Hojun Shin, Kambiz Sadeghi, Jongchul Seo

**Affiliations:** 1 Department of Packaging and Logistics, Yonsei University, 1 Yonseidae-gil, Wonju-si 26493, Gangwon State, Republic of Korea; beancolor@naver.com (J.L.); ghwns0310@naver.com (H.S.); 2 School for Engineering of Matter, Transport and Energy, Arizona State University, 501 E Tyler Mall, Tempe, AZ 85287, USA; kambiz_sadeghi@ymail.com

**Keywords:** ClO_2_ self-release, chlorine dioxide, sterilizing agent, postharvest life, fresh-food packaging

## Abstract

Chlorine dioxide (ClO_2_) is a powerful sterilizing agent that is widely used to prevent the spoilage of fresh foods during delivery and storage. However, its practical applications are hindered by a short sterilization duration, complex deployment processes, and high treatment costs. To address these challenges, an innovative ClO_2_ self-releasing sachet was developed, which was specifically designed for use in retail and wholesale markets. The sachet utilizes polyether block amide (PEBAX^®^) as a hydrophilic polymer to facilitate the dissociation of sodium chlorite (NaClO_2_) and citric acid (CA), which generates ClO_2_. A PEBAX/CA composite film was coated onto kraft paper to construct the sachet. This design extended the ClO_2_ release period to over 3 d, with a controllable release rate being achieved by adjusting the concentrations of NaClO_2_ and CA. In practical tests, the sachets inhibited fungal growth by >50% over 14 d at 20 °C within a corrugated box. Furthermore, they preserved the quality of the cherry tomatoes for 16 d during storage. These results demonstrate that the newly developed sachet offers an economical and user-friendly solution for fresh-food packaging, effectively preserving product quality.

## 1. Introduction

The demand for fresh foods, such as vegetables and fruits, has risen significantly due to an increasing consumer focus on health and wellness. In 2022, the global fresh-food market was valued at USD 3.2 trillion, and it is projected to grow to USD 4.8 trillion by 2032, reflecting a compound annual growth rate (CAGR) of 4.2% [1]. However, this surge in demand has also led to a corresponding increase in food waste during delivery and storage. One primary cause of food waste is quality deterioration, which is often driven by the presence of moisture in the packaging material [2]. Excessive moisture fosters microbial growth, which leads to postharvest spoilage issues such as textural deformation, unpleasant odors, and rotting caused by cell breakdown [3,4]. To mitigate these challenges, sterilization technologies are increasingly employed to preserve food quality during storage and transportation.

Chlorine dioxide (ClO_2_) has emerged as a powerful sterilizing agent approved by the US Food and Drug Administration (FDA) for use with fresh foods [5,6,7]. It is widely regarded as a safer alternative to chlorine, which produces harmful halogenated disinfection byproducts such as trihalomethanes and haloacetic acids [8,9]. This transition enhances consumer safety by minimizing the potential health risks arising from corrosion or residues during sterilization. To support its viability, the European Food Safety Authority (EFSA) has conducted safety assessments for the use of slow-releasing gaseous ClO_2_ in cold food storage environments, further underscoring its potential for broader applications in the food industry [10]. Food-grade ClO_2_ derivatives, both in gaseous (ClO_2(g)_) and aqueous (NaClO_2(aq)_) forms, are utilized for sterilization through two main methods, namely liquid application and gaseous dispersion. Liquid application involves spraying or immersing fresh foods in aqueous ClO_2_ solutions. However, these methods increase packaging moisture levels and necessitate specialized, costly equipment [11,12]. Additionally, liquid-based sterilization systems often fail to achieve uniform coverage of the produce surfaces, thereby limiting their effectiveness. In contrast, gaseous ClO_2_ can be uniformly distributed over the produce, rendering it a more effective solution for sterilization [13]. Recent advancements have focused on developing sterilization techniques that leverage ClO_2_ gas to achieve superior and consistent results compared to those obtained using liquid-based methods. Notably, Trinetta et al. demonstrated that minimal ClO_2_, chlorite, chlorate, and chloride residues were found on the surfaces of various fresh foods (including tomatoes, oranges, apples, strawberries, lettuce, bean sprouts, and cantaloupe), with values being well below the acceptable levels prescribed by the Environmental Protection Agency (EPA) for drinking water, thereby indicating a negligible consumer risk [14]. Despite these advantages, ClO_2_ gas faces significant practical challenges. For example, it is not possible to compress or commercially store high concentrations of ClO_2_ gas (>10%) due to its explosive nature [15]. This limitation necessitates expensive equipment and specialized processing facilities, which drastically increase costs and restrict scalability. Furthermore, ClO_2_ gas has a low boiling point of 11 °C, which limits its stability and reduces its efficacy over time, rendering it less suitable for long-distance food transport [16]. These challenges highlight the need for further innovation to enhance the practical utility of ClO_2_ gas sterilization systems in the food packaging industry.

To address the above obstacles, novel approaches have been explored to achieve controlled ClO_2_ gas release from packaging, including encapsulation techniques and ClO_2_ solution pouches [17,18]. These innovations have extended the gas-release period to 6 d and have prolonged its sterilization effectiveness to 11 d. However, these methods also present drawbacks, such as complex manufacturing processes and residual explosion risks, ultimately limiting their feasibility for industrial-scale deployment. Consequently, additional research and development are necessary to overcome these limitations and enable the widespread application of ClO_2_ gas sterilization systems in the food industry.

Previously, our research group prepared an innovative smart sachet capable of releasing ClO_2_ gas in a controlled manner, which was activated by the moisture produced by the packaged products. This system leverages a combination of hydrophilic polymers, carboxylic acids, and sodium chlorite (NaClO_2_) to regulate ClO_2_ gas production upon exposure to water. The mechanism involves three key stages, namely water absorption, proton generation and diffusion, and ClO_2_ generation and release. Firstly, in terms of water adsorption, the hydrophilic polymer matrix in the sachet efficiently absorbs water molecules from the surrounding environment under humid conditions. This process occurs on both the surface and within the matrix of the polymer material, ensuring an optimal water intake. Secondly, in the context of proton generation and diffusion, after water molecules penetrate the sachet, they interact with the carboxylic acid species embedded within the polymer matrix. This interaction triggers the dissociation of the carboxylic acid, releasing hydrogen ions (proton, H⁺). These species subsequently diffuse through the water-saturated polymer network, gradually migrating toward the embedded NaClO_2_ powder. Thirdly, regarding ClO_2_ generation and release, when the diffused H⁺ ions encounter the NaClO_2_ particles, a chemical reaction occurs, leading to the production of ClO_2_ gas. The generated ClO_2_ is then released into the surrounding environment in a controlled manner, effectively targeting areas with increased moisture levels. This controlled release mechanism allows for the targeted application of ClO_2_ gas in response to moisture exposure, as described by Huang et al. [19]:

(1) $$-COOH↔-COO^{-}+H^{+}$$

(2) $$5ClO_{2}^{-}+4H^{+}\to 4{ClO}_{2}↑+\;Cl^{-}+2H_{2}O$$

Notably, the aqueous medium facilitates a rapid reaction due to the efficient dissociation of solid NaClO_2_ into Na^+^ and ClO_2_^−^ in the presence of moisture, enabling a burst release of ClO_2_ [20]. Polyether-b-amide (commercially known as PEBAX^®^), a block copolymer consisting of a rigid polyamide (PA) segment and a flexible polyether (PE) segment, is an excellent material for enhancing this dissociation [21]. More specifically, PEBAX^®^ MH 1657 (PEBAX) is widely used in various applications, including active molecular carriers and gas-separation membranes [22]. Furthermore, it demonstrates outstanding gas permeability and moisture retention capabilities due to the presence of hydroxyl groups in its polyethylene oxide (PEO) segment, thereby effectively maintaining adequate moisture levels within the polymer matrix. In addition, citric acid (CA), which is an FDA-approved natural organic tricarboxylic acid, has been considered a promising material for food packaging due to its colorless, odorless, and mildly acidic properties [23,24].

In this study, CA is incorporated into a composite film to promote and regulate ClO_2_ generation. This ClO_2_-releasing sachet consists of PEBAX and CA (PEBAX/CA) composite films combined with NaClO_2_ powder. Kraft paper serves as the structural layer, providing mechanical strength and preventing leakage after the absorption of moisture by the composite film. This innovative sachet has a simple, compact form that eliminates the requirement for additional equipment or costs, in contrast to conventional ClO_2_ treatment methods. Moreover, it is expected that this system will address the short-lived antimicrobial effects that are typical of traditional ClO_2_ treatments by utilizing the moisture that is naturally released from the freshly harvested produce as a trigger. The physical properties (i.e., ductility, thermal stability, and water sorption ability) of the prepared PEBAX/CA composite film are evaluated using varying CA concentrations (0, 5, and 10 wt.%), and the feasibility of employing this smart sachet for real-world applications is assessed based on ClO_2_ gas-release and storage tests using cherry tomatoes. It is hypothesized that the smart ClO_2_-releasing sachet will extend the shelf life and preserve the quality of cherry tomatoes during storage.

## 2. Results and Discussion

### 2.1. Characterization of the Composite Films

#### 2.1.1. Fourier Transform Infrared (FT-IR) Spectroscopy

**Figure 1:**
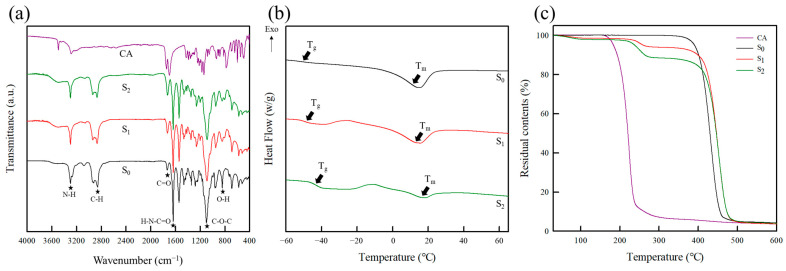
Chemical structures and thermal properties of pure PEBAX and the PEBAX/CA composite films: (**a**) ATR-FT-IR spectra, (**b**) DSC curves, and (**c**) TGA curves.

The chemical structures of CA, the pure PEBAX film (S_0_), and two PEBAX/CA composite films (S_1_ and S_2_) were analyzed using Fourier transform infrared (FT-IR) spectroscopy, as detailed in Figure 1a and Table 1. For CA, several characteristic bands were identified. For example, the signals at 3495 and 3292 cm^−1^ corresponded to the O–H stretching vibrations of the hydroxyl groups (–OH), while the band at 1699 cm^−1^ was attributed to the C=O stretching vibrations of the carboxylic acid groups (–COOH) [25,26]. Additionally, the peak at 1742 cm^−1^ was assigned to interference that results from the close proximity of carboxyl groups within a diacid [27,28]. The pure PEBAX film (S_0_) exhibited distinct characteristic peaks intrinsic to PEBAX, including a peak at 845 cm^−1^ corresponding to the O–H stretching vibrations of the hydroxyl groups, a peak at 1638 cm^−1^ corresponding to the H–N–C=O moiety, and a peak at 3297 cm^−1^ attributed to the N–H stretching vibrations. Furthermore, the peak at 1100 cm^−1^ was assigned to the C–O–C stretching vibrations within the ether groups of the PEO segment [29,30,31,32,33]. Adsorption bands were also observed at 1729 and 2896 cm^−1^, corresponding to the C=O stretching vibrations of the carbonyl groups and the C–H bending vibrations, respectively [34,35,36].

S_1_ and S_2_ exhibited characteristic peaks similar to those of S_0_ due to the presence of PEBAX. However, the peak at 1735 cm^−1^ (C=O) in the PEBAX/CA spectra shifted to 1731 cm^−1^ and increased in intensity upon increasing the CA content. This shift can be attributed to the esterification between PEBAX and CA. More specifically, the carboxyl groups of CA interact with the hydroxyl groups of PEBAX, forming linkages containing carbonyl groups (C=O) [28]. Similar results were reported by Huang et al., who observed an increase in the intensity of the C=O group signal upon esterification between the polyester hydroxyl groups and the carboxyl groups of CA within crosslinked networks [37]. In addition, the peak observed at 845 cm^−1^ in the PEBAX/CA spectra decreased as the CA content was increased, which is also associated with esterification. Similarly, Seligra et al. demonstrated that the number of O–H groups decreased upon esterification between the carboxyl groups of CA and the hydroxyl groups of glycerol in crosslinked networks [38]. The current study integrates CA into PEBAX to develop a platform where CA serves as a source of protons (H^+^). To preserve the proton-generating capacity of CA, the PEBAX/CA composite films were not subjected to additional treatment (e.g., annealing), thereby ensuring that the deprotonation capacity of CA remained intact within the composite film matrix. Consequently, the interactions between PEBAX and CA maintained the functional integrity of both components.

#### 2.1.2. Scanning Electron Microscopy (SEM)

**Figure 2:**
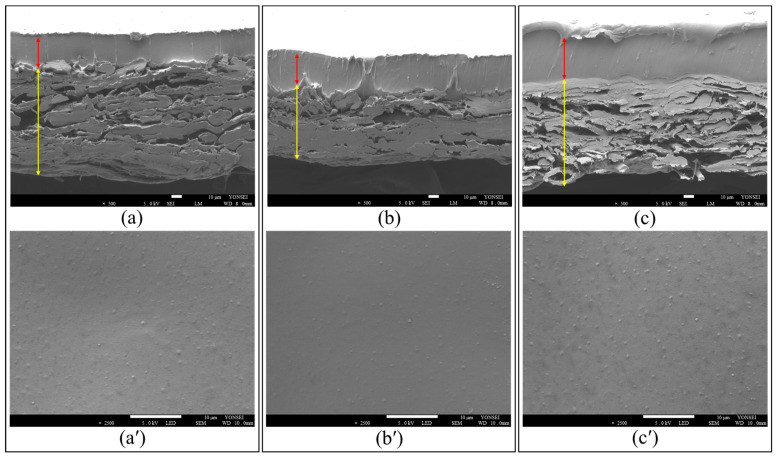
Cross-sectional and surface SEM images of the (**a**,**a′**) S_0_-coated paper, (**b**,**b′**) S_1_-coated paper, and (**c**,**c′**) S_2_-coated paper. The yellow line indicates the kraft paper (structural) layer, while the red line indicates the PEBAX or PEBAX/CA (coating) layer.

The physical properties of a coating layer, including its uniformity and thickness, are critical in determining its performance [39,40]. These morphological properties, therefore, directly influence key characteristics of the developed sachet, including its tensile strength, moisture adsorption capabilities, and ClO_2_ release kinetics. To better understand these effects, the top surfaces and cross-sections of the S_0_, S_1_, and S_2_ specimens were analyzed using scanning electron microscopy (SEM), as shown in Figure 2. It was revealed that the coating layers of the sachets adhered well to the structural layer, maintaining similar thicknesses regardless of the CA content. This was primarily attributed to the presence of flexible PEO segments in PEBAX, which support consistent film formation [41]. Notably, the addition of CA at lower temperatures did not interfere with the coating process, as the PEBAX solution retained its film-forming ability. Furthermore, all the samples exhibited smooth and uniform surfaces without any signs of aggregation or voids. These findings suggest that the mechanical strength, barrier properties, and gas-release performances of the composite films and sachets can be effectively modulated by tuning the chemical composition and morphological characteristics of the coating layer.

#### 2.1.3. Thermal Properties

The thermal properties of composite films are influenced by the reactivities of their components in addition to their miscibilities and crosslinking degrees [42]. Differential scanning calorimetry (DSC) and thermogravimetric analysis (TGA) were performed to determine the thermal properties of CA, the pure PEBAX film (S_0_), and the two PEBAX/CA composite films (S_1_ and S_2_), as detailed in Figure 1b and Table 2.

PEBAX is known to exhibit two distinct melting temperatures (T_m_) owing to its polyphase-separated structure. The lower T_m_ (13–17 °C) corresponds to the soft PEO segment, while the higher T_m_ (200–205 °C) is associated with the hard PA segment [43]. This unique thermal behavior enables PEBAX to remain in its solid form at room temperature. The high melting point and crystallinity of the PA segments impart structural stability, while the microphase-separated morphology provides a balance between flexibility and rigidity. In this study, it was observed that the thermal decomposition of CA occurred at 180 °C, which influenced the melting behavior of PEBAX, leading to a higher T_m_ and causing an overlap. To mitigate this overlap, DSC measurements were conducted in the temperature range of −70 to 70 °C. It was found that S_0_ exhibited a glass-transition temperature (T_g_) of −50.8 °C and a T_m_ of 13.7 °C. In contrast, the T_g_ values of S_1_ and S_2_ increased to −49.3 and −42.9 °C, respectively, as the CA content was increased. This shift in T_g_ can be attributed to a physical crosslinking reaction between the hydroxyl groups of PEBAX and the carboxyl groups of CA, as confirmed by the FT-IR analysis. In addition, the T_m_ values of the S_1_ and S_2_ specimens increased to 15.4 and 17.7 °C, respectively, upon increasing the CA content. This can be accounted for by considering the enhanced size and stability of the PEBAX/CA crystal structure due to the formation of additional crosslinked networks at higher CA concentrations [44]. However, the melting enthalpy (ΔH_m_) was significantly reduced as the CA content was increased, likely due to the restricted ability of the PEBAX molecular chains to rearrange and form crystalline segments within the crosslinked structure [45].

The TGA was performed to evaluate the thermal stabilities of the pure PEBAX film (S_0_) and the PEBAX/CA composite films (S_1_ and S_2_), as presented in Figure 1c. The pure CA and S_0_ samples exhibited single-step decomposition patterns, occurring in the temperature ranges of 180–270 and 350–460 °C, respectively. In contrast, S_1_ and S_2_ demonstrated two-step decomposition patterns, wherein the first step (T1st, 220–270 °C) corresponded to the decomposition of CA, and the second step (T2nd, 350–490 °C) corresponded to the decomposition of PEBAX [46,47].

Both the thermal decomposition steps of S_1_ and S_2_ were shifted to higher temperatures than those of the pure CA and PEBAX samples, indicating that thermal stability increased. This phenomenon is potentially due to the strong chemical interactions within the composite matrix, which arose mainly through crosslinking. Notably, crosslinking enhances intermolecular interactions and restricts molecular mobility, thereby increasing thermal stability. Furthermore, the increased number of chemical bonds in the crosslinked network requires additional energy for thermal degradation to occur [48,49]. Notably, no significant differences were observed in the thermal decomposition steps of S_1_ and S_2_ despite variations in the CA content. Additionally, the composite films did not undergo decomposition before reaching the maximum manufacturing temperature (80 °C). This finding indicates that the CA content can be adjusted in the composite films without compromising the thermal stability.

#### 2.1.4. Mechanical Properties

**Figure 3:**
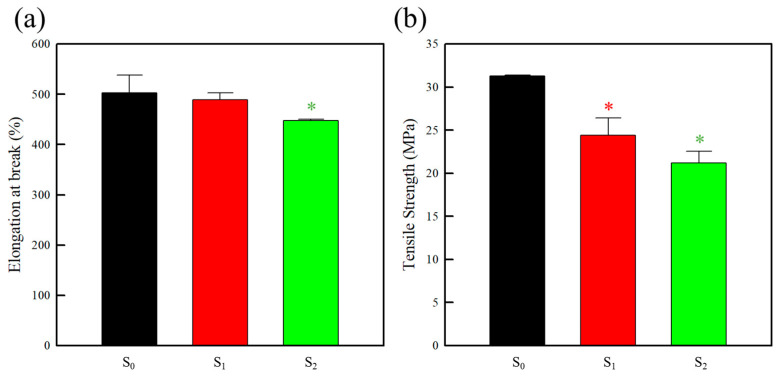
Mechanical properties of the pure PEBAX and PEBAX/CA composite films: (**a**) elongation at break and (**b**) tensile strength. The error bars indicate the standard deviations of the reported measurements. The values marked with the asterisk (*) show statistically significant differences compared to the S_0_ sachet. (*p* < 0.05).

The influence of the CA content on the mechanical properties of the PEBAX and PEBAX/CA composite films was subsequently evaluated using a universal testing machine (UTM). According to the technical datasheet, PEBAX exhibits an excellent mechanical performance, characterized by a tensile strength exceeding 13 MPa and an elongation at break >50%. This performance is attributed to its unique structure, which combines a rigid PA segment with a flexible PEO segment [50]. In the present study, the tensile strength and elongation at break of the S_0_ specimen were determined to be 31.3 MPa and 502.3%, respectively. The addition of CA led to a slight reduction in the elongation at break of the composite film and a more pronounced decrease in the tensile strength, as illustrated in Figure 3. This behavior can be attributed to the crosslinking effect of CA, which reduces the flexibility of the polymer matrix [42]. Despite the reduced tensile strength, the introduction of CA preserved the elongation behavior of the material, which remained >400%. As a result, the CA-containing composite films (S_1_ and S_2_) demonstrate strong mechanical properties, rendering them suitable for use as flexible packaging materials or coating layers.

#### 2.1.5. Water Sorption

**Figure 4:**
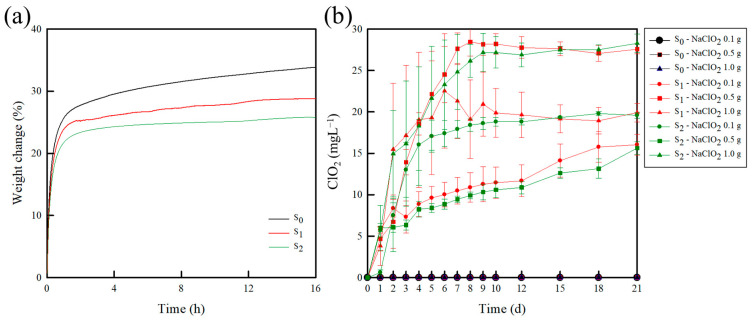
(**a**) Water sorption isotherms of the PEBAX/CA-coated paper. (**b**) Concentrations of ClO_2_ released from the sachets. The error bars indicate the standard deviations of the reported measurements.

Moisture plays a crucial role as a catalyst in producing ClO_2_^−^ (from NaClO_2_) and H^+^ (derived from CA), which serve as precursors for ClO_2_ production in the current study. As shown in Figure 4a, all the samples exhibit a rapid weight increase during the initial 90 min, followed by a slower yet continuous weight gain. The final water uptake capacities were 33.8, 28.8, and 25.7 wt.% for the S_0_, S_1_, and S_2_ specimens, respectively. Considering that the water uptake capacity is influenced by the chemical affinity of the polymer matrix for water (i.e., its hydrophilicity) [51], the reduced water uptake capacities of the S_1_ and S_2_ samples were partly attributed to the introduction of CA, which reduces the number of hydrophilic hydroxyl groups [52,53]. This observation aligns with the FT-IR results, wherein a reduction in the hydroxyl group intensity was noted. Despite this, the samples maintained a high water sorption capacity exceeding 20 wt.%. This suggests that the developed sachets likely absorbed sufficient moisture to generate the ClO_2_ precursors (ClO_2_^−^ and H^+^), which are essential for sustained gas release.

### 2.2. Release Test of ClO_2_ from the Sachets

The release of ClO_2_ from the developed sachets can be influenced by several factors, including temperature, humidity levels, degree of light exposure, NaClO_2_ concentration, and CA content. To evaluate the controllability of ClO_2_ release based on the contents of CA and NaClO_2_, release tests were conducted using sachets containing varying amounts of NaClO_2_, as shown in Figure 4b and Table 3. The S_0_ samples did not exhibit any detectable release of ClO_2_ irrespective of the NaClO_2_ concentrations. In contrast, all the S_1_ and S_2_ samples consistently release ClO_2_ over a period of 6–9 d. The CA content had a slight impact on the ClO_2_ release rate, while an increase in the NaClO_2_ content led to a more pronounced effect. More specifically, higher NaClO_2_ content increased the initial release rate and prolonged the release duration. This behavior can be attributed to an increase in the production of ClO_2_^−^, which subsequently increased the ClO_2_ release rate. Furthermore, the total concentrations of ClO_2_ released were determined to be 16.1, 19.9, and 27.6 mg L^−1^ for the sachets containing 0.1, 0.5, and 1.0 g of NaClO_2_, respectively. These results indicate that ClO_2_ release from the prepared sachets can be tailored by adjusting the NaClO_2_ and CA contents, thereby offering a versatile solution for various applications. It has previously been reported that excessive ClO_2_ exposure can compromise the product quality by causing skin cracking, bleaching, and undesirable changes in the sensory attributes of the product, such as the aroma and taste, ultimately reducing its marketability [54,55]. Conversely, insufficient ClO_2_ concentrations within the packaging may fail to achieve an effective antimicrobial action. Based on these considerations, the sachet containing 0.1 g of NaClO_2_, which exhibited the lowest ClO_2_ release rate, was selected for subsequent storage tests. 

As described above, the release of ClO_2_ in the current system is moisture-triggered, and as a result, preliminary studies demonstrated that the sachets stored under dry conditions did not release ClO_2_ [18]. Therefore, to achieve a moisture-responsive system, the materials were optimized. More specifically, PEBAX plays a crucial role by entrapping moisture, which facilitates the dissociation of CA and NaClO_2_. This reaction produces ClO_2_ precursors, including ClO_2_^−^ and H^+^ ions. The soft segment of PEBAX enhances the diffusion of permeants through its matrix, allowing the precursors to interact and generate ClO_2_ gas effectively.

### 2.3. Storage Test in a Plastic Box

The applicability of the S_0_, S_1_, and S_2_ sachets was evaluated through a storage test conducted at 25 °C for 16 d. Cherry tomatoes were used as the test produce, and their quality was assessed based on parameters such as the pH, total soluble solid (TSS) content, firmness, and visual appearance. These assessments provided insights into the efficacies of the sachets and their suitability for use in the packaging of fresh foods [56,57]. The cherry tomatoes were packaged in a standard plastic clamshell box, a commonly used container for fresh produce. The results of the two-way ANOVA of pH, TSS, and firmness are listed in Table 4.

**Figure 5:**
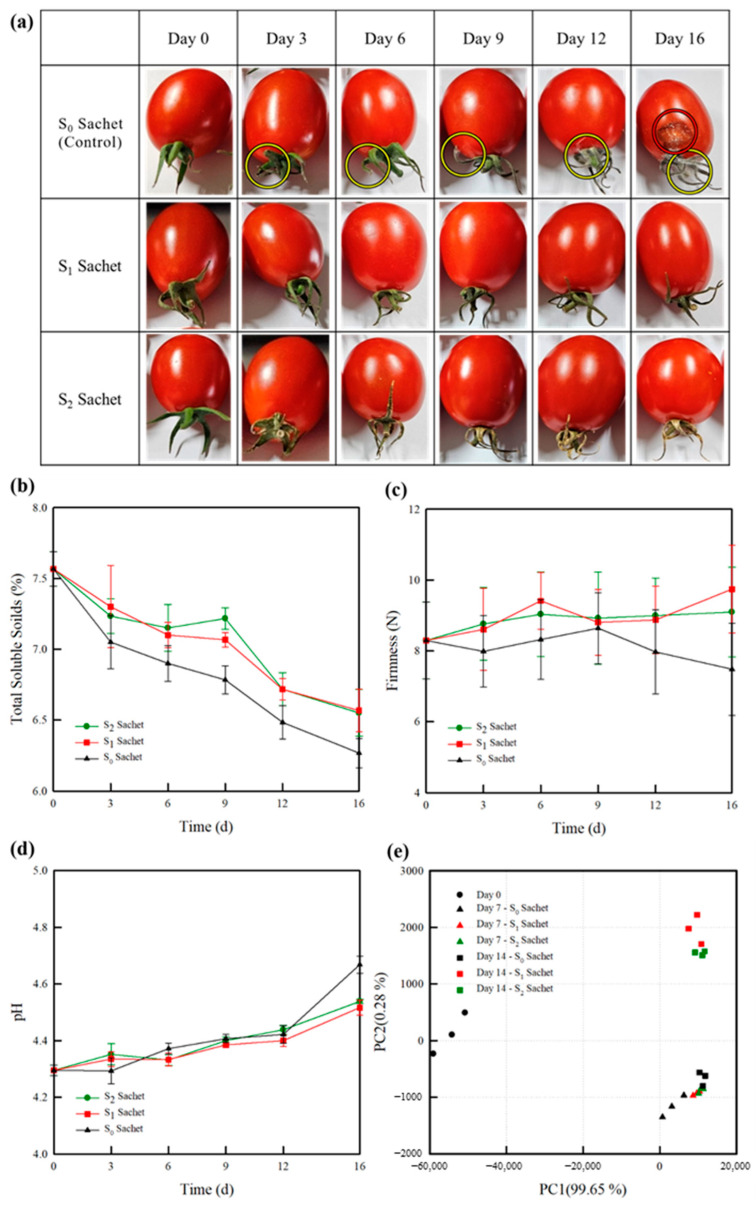
Quality parameters of the cherry tomatoes during storage: (**a**) visual appearance, (**b**) TSS content, (**c**) firmness, and (**d**) pH. (**e**) Corresponding PCA results. The error bars indicate the standard deviations of the reported measurements.

Visual appeal is a critical determinant of consumer preference in the fresh-food market. As shown in Figure 5a, the control samples (without ClO_2_ treatment) show signs of fungal growth by day 3 (yellow circle) due to microbial activity and are noticeably spoiled by day 16 (red circle). In contrast, the cherry tomatoes treated with the developed ClO_2_ sachets remain free of fungal growth and spoilage throughout the 16 d period. However, the calyxes of the cherry tomatoes packaged with the S_2_ sachet begin to show bleaching as early as day 3. This phenomenon can be attributed to the degradation and decolorization of chlorophyll caused by the strong oxidizing properties of ClO_2_ [58,59]. Based on these observations, the S_1_ sachet was identified as the optimal choice under the given storage conditions, as it effectively maintained the quality of the cherry tomatoes without compromising appearance.

As shown in Figure 5b, the TSS contents of all the samples decrease over the storage period, primarily due to the ripening of the cherry tomatoes. By the 16th day, the TSS values for the control and treated cherry tomatoes decline from an initial value of 7.7% to 6.15, 6.67, and 6.57% for the S_0_, S_1_, and S_2_ sachets, respectively. The cherry tomatoes with S_0_ exhibit a more rapid reduction in the TSS content compared to the samples treated with the developed sachets, which maintain higher TSS levels. This behavior aligns with the known effects of increased microbial activity and increased fruit respiration, which contribute to the reduced TSS values. These findings also correspond to observable changes in the visual quality of the S_0_ sample [60].

While the S_0_ group experienced a steady decline in firmness (Figure 5c), the S_1_ and S_2_ samples showed good preservation of their initial firmness for up to 9 d, followed by a more rapid decrease thereafter. In terms of pH, the S_0_ sample exhibited a significant increase, reaching 4.67 by day 16, while the pH levels of the S_1_ and S_2_ samples increased to a lesser extent (Figure 5d). This result is consistent with previous reports stating that the pH levels of tomatoes increase with respiration, ripening, and increased storage times [61].

A principal component analysis (PCA) was conducted to show the differences among the samples based on data obtained using an electronic nose (e-nose), as presented in Figure 5e. The total contributions of the first principal component (PC1) and second principal component (PC2) were 99.7 and 0.3%, respectively. In general, the PCs effectively represent the original data when their cumulative contribution exceeds 85% [62]. Therefore, the PC1 value provides sufficient information for classifying fragrance patterns. On day 0, the cherry tomato samples with S_0_ exhibited PC1 values between −60,000 and −50,000, while on days 7 and 14, the corresponding values were between 0 and 15,000. This shift can be attributed to the postharvest ripening process characteristics of cherry tomatoes. In contrast, the differences were less significant for the cherry tomatoes stored in the presence of the S_1_ and S_2_ sachets. This observation confirms that no additional odor was produced by the ClO_2_ released from the sachets. Moreover, the obtained results indicate that the release of ClO_2_ influenced the respiratory and enzymatic activities, thereby delaying biological degradation and preserving the overall product quality [63]. The two-way ANOVA analysis demonstrated that both the treatment (citric acid concentration) and storage time, as well as their interaction, had statistically significant effects (*p* < 0.001) on the ClO₂ release and quality parameters (TSS, firmness, and pH) of the cherry tomatoes. These findings suggest that the combination of citric acid incorporation and storage conditions plays a critical role in modulating the postharvest quality of the produce.

### 2.4. Storage Test in a Corrugated Box

**Figure 6:**
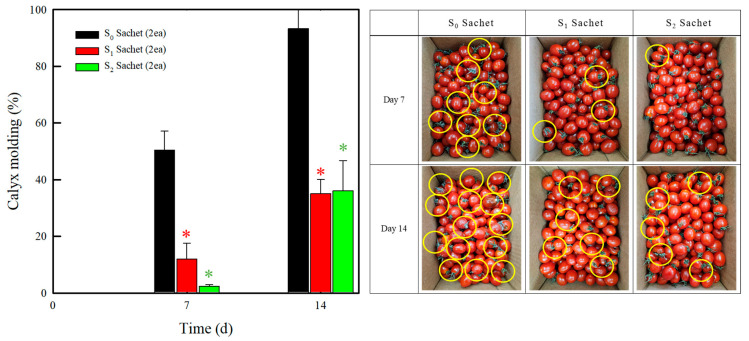
Fungal growth rates of the cherry tomatoes and their visual appearances during storage. The error bars indicate the standard deviations of the reported measurements. The values marked with the asterisk (*) show statistically significant differences compared to S_0_ sachet. (*p* < 0.05).

To evaluate the sterilization effects of the sachets in commercial storage conditions, tests were conducted using larger-scale setups, including kilogram units and corrugated cardboard boxes, as shown in Figure 6. In the S_0_ group, fungal growth was observed in 50.5 and 93.3% of the total cherry tomato population on days 7 and 14, respectively. Conversely, the cherry tomatoes treated with the S_1_ and S_2_ sachets demonstrated more than a 50% reduction in the occurrence of moldy tomatoes compared to the S_0_. However, the decolorizing effect of the S_2_ sachets observed during the storage tests performed in plastic boxes was not evident in the commercial corrugated boxes. This suggests that the larger storage container and the greater quantity of cherry tomatoes (500–2000 g) ensured an optimal distribution of the released ClO_2_ to the stored produce. These results clearly demonstrate that the developed sachets can serve as an efficient and convenient alternative to conventional sterilization methods, significantly maintaining the quality of fresh products.

Overall, these ClO_2_-releasing sachets exhibited outstanding mechanical properties, efficient gas-release performances, and effective quality-retention capabilities during the storage of cherry tomatoes. These results validate that the smart ClO_2_-releasing sachets can preserve the quality of cherry tomatoes under real-world storage conditions.

## 3. Materials and Methods

### 3.1. Materials

PEBAX^®^ MH 1657 was procured from Arkema Co., Ltd. (Paris, France). Ethanol (≥99.5%) was supplied by Daejung Chemicals and Metals Co., Ltd. (Siheung, South Korea). CA (≥99.5%) and NaClO_2_ (≥80%) were purchased from Merck Co., Ltd. (Seoul, South Korea). Deionized (DI) water was utilized throughout the study, and all the chemicals were used as received without further purification.

### 3.2. Preparation of the PEBAX/CA Composite Films and Sachets

**Figure 7:**
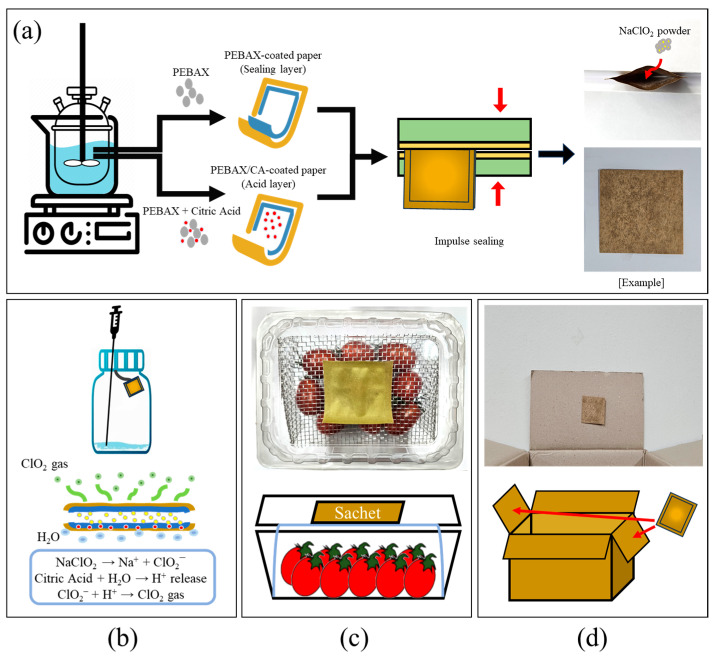
Schematic representations showing the (**a**) manufacture of the smart ClO_2_-releasing sachets, (**b**) gas-release test performed on the sachet, (**c**) storage test performed in a plastic clamshell box, and (**d**) storage test performed in a corrugated box.

The composite films for the smart ClO_2_-releasing sachets were fabricated using a solution-casting method. Initially, PEBAX (13 wt.%) was dissolved in a 70:30 wt.% ethanol/water mixture. This solution was stirred at 80 °C and 200 rpm for 2 h. Separately, CA (5 and 10 wt.%) was dissolved in DI water at room temperature (25 °C). After heating the PEBAX solution to 50 °C, the CA solution was added, followed by stirring for 10 min. The mixture was then cast onto kraft paper using a bar-type automatic coating film applicator (KIPEA E&T Co., Ltd., Hwasung, South Korea). To prevent undesirable reactions, such as crosslinking or poor film formation, CA was introduced at a reduced temperature (50 °C) and with minimal stirring durations. The resulting solutions were cast onto glass substrates to produce neat PEBAX/CA composite films. A separate sealing layer was prepared using only PEBAX, following the same procedure. Both the pure PEBAX films and the PEBAX/CA composite films were maintained at a uniform thickness of 100 μm, while the kraft paper thickness was controlled at 170 μm. The prepared samples were labeled S_0_, S_1_, and S_2_ based on their CA contents (Table 5). Each sample was analyzed in its film, coated paper, and sheet forms, sharing the same sample codes across all the experiments. The coated paper was manually cut into 5 cm × 5 cm pieces and sealed using an impulse adhesive sealer (Iss 350−10, Gasungpack, Gwangju, South Korea). To complete the preparation of the sachets, the NaClO_2_ powder (0.1 g) was placed inside the sealed structure, as depicted in Figure 7a.

### 3.3. Characterization

#### 3.3.1. Properties of the Composite Films

To evaluate the chemical structures of the composite films, an FT-IR spectroscopy (65 FT-IR, PerkinElmer Co., Waltham, MA, USA) was conducted in the wavenumber range of 4000–400 cm^−1^ using the attenuated total reflection (ATR) mode with a diamond/ZnSe crystal. The morphologies of the top and cross-sectional surfaces of the PEBAX/CA-coated paper were observed using field-emission SEM (FE-SEM, JEOL-7800F, JEOL Co., Ltd., Tokyo, Japan) at an acceleration voltage of 5 kV and a working distance of 10 mm. Prior to the SEM imaging, all the samples were coated with a thin Pt layer to enhance the conductivity and image quality. The thermal properties of the PEBAX/CA composite films were examined using DSC (Q10, TA Instrument Co., Ltd., New Castle, DE, USA). For this purpose, the samples were heated from −70 to 230 °C at a rate of 10 °C/min under a nitrogen atmosphere with a flow rate of 20 mL/min. The thermal stabilities and weight loss behaviors of the composite films were evaluated using TGA (4000 TGA, PerkinElmer Co., Ltd., Waltham, MA, USA). These measurements were carried out in a nitrogen atmosphere over a temperature range of 30–800 °C and at a heating rate of 10 °C/min.

The mechanical properties of the PEBAX/CA composite films were measured using a UTM (QM 100 T, Qmesys Co. Ltd., Uiwang, South Korea). Dumbbell-shaped specimens (Type IV) were prepared according to the ASTM D638-14 standard testing method [64].

The water sorption behavior of the PEBAX/CA-coated paper was analyzed using a dynamic vapor sorption (DVS) instrument (DVS Intrinsic, Surface Measurement Systems, London, UK) equipped with an SMS UltraBalance™ system that offered a mass resolution of ± 0.1 μg. The experiments were conducted at a relative humidity (RH) of 95% for 24 h on the samples with an average mass of 5 mg. Prior to analysis, the samples were dried under a dry nitrogen flow at 25 °C and 0% RH for 2 h.

#### 3.3.2. Gas-Release Tests

A simulated release test was designed to evaluate the release of ClO_2_ gas from the sachets. The test setup, shown in Figure 7b, involved a 1.6 L glass jar containing water (3 mL) to replicate the moisture generated by the respiration of fresh foods. In the closed system, it was assumed that the majority of the released ClO_2_ gas dissolved in the water. To determine the ClO_2_ concentration in the packaging, the solution was analyzed using a UV–vis spectrophotometer (UV-2600, Shimadzu, Tokyo, Japan) at a wavelength of 358 nm [65,66]. The ClO_2_ solution was drawn into a 10 mL syringe and transferred into a quartz cuvette for the absorbance measurements. The concentration of ClO_2_ gas in the packaging was quantified using the Beer–Lambert law, as defined in Equation (3):

(3) $$\left[{\mathrm{ClO}}_{2}\right]\left(M\right)=A/l\varepsilon$$

where *A*, *l*, and ε represent the absorbance of the sample, the path length of the cuvette (in cm), and the molar absorptivity of ClO_2_, respectively. The molar absorptivity (ε) of ClO_2_ in water is 1250 L mol^−1^ cm^−1^ [67]. The result derived from this equation was then multiplied by the molecular weight of ClO_2_ (67,450 mg mol^−1^) to express the concentration in terms of mg L^−1^ ClO_2._

#### 3.3.3. Storage Tests in a Plastic Box

Storage tests were conducted to evaluate the sterilization effects of the sachets on the food quality using cherry tomatoes obtained from a local farm in Wonju, South Korea. The test was carried out at 25 °C for 16 d. As shown in Figure 7c, the cherry tomatoes (150 g) were placed in a 1.6 L plastic clamshell box, each incorporating either sachet S_0_, S_1_, and sachet S_2_. The pH, TSS content, and sample firmness were measured in each case. The pH was determined using a digital pH meter (Hanna Instrument, Woonsocket, RI, USA), while the TSS contents were obtained using a digital Brix refractometer (PAL–3, Atago Co., Ltd., Tokyo, Japan). The firmness was analyzed using a fruit hardness tester (FR–5105, Lutron Electronic Enterprise Co., Ltd., Taipei, Taiwan), which measured the maximum compressive force applied by a 3 mm diameter cylindrical probe, recorded in Newtons (N). The PCA data were analyzed using an e-nose (Heracles NEO Electronic Nose, Alpha MOS Co., Ltd., Toulouse, France). Prior to analysis, the samples were homogenized in sterile stomacher filter bags using a stomacher (BagMixer^®^, Interscience, Saint Nom, France) for 2 min to ensure uniformity.

#### 3.3.4. Large-Scale Storage Test Using Corrugated Boxes

A separate storage test was conducted using commercial packaging to simulate actual distribution conditions. Cherry tomatoes (2 kg), harvested on the same day, were packed in a standard corrugated box commonly used at the farm. S_0_, S_1_, and S_2_ sachets were, respectively, mounted on the top inner surfaces of separate boxes. The boxes were stored at room temperature (25 °C) and 60% RH for 14 d. On days 7 and 14, the boxes were opened to assess visible changes in the cherry tomatoes, including fungal growth and bleaching. These changes were evaluated through visual inspection by trained researchers with extensive experience in postharvest quality and spoilage assessment, using standardized observation protocols to ensure consistency and reliability.

#### 3.3.5. Statistical Analysis

All the experimental data were expressed as the mean ± standard deviation (SD) of at least three independent replicates. For thermal and mechanical properties of the sachet and calyx molding, one-way ANOVA was conducted to determine the statistical differences among the S_0_, S_1_, and S_2_. In contrast, the data regarding ClO₂ release and the quality parameters of cherry tomatoes (including TSS, firmness, and pH) were analyzed using two-way ANOVA to assess the effects of treatment (citric acid concentration), storage time, and their interaction. Duncan’s multiple range test was applied for post hoc comparisons when significant differences were detected (*p* < 0.05). All the statistical analyses were performed using the SPSS software (IBM SPSS statistics 27.0, SPSS Inc., Chicago, IL, USA).

## 4. Conclusions

In this study, a ClO_2_-releasing sachet was developed to maintain the quality of fresh foods, and its potential as an innovative alternative to conventional sterilization technologies in the food packaging industry was demonstrated. The physical and morphological properties of the prepared sachet were systematically analyzed, and its applicability was evaluated. It was found that the incorporation of CA into the PEBAX^®^ matrix resulted in crosslinking. As the CA content was increased, the thermal stability increased, whereas the tensile strength, elongation at break, and water sorption capability decreased. Furthermore, controlled ClO_2_ release was successfully achieved, and the quantity of released gas could be finely tuned by adjusting the concentrations of NaClO_2_ and CA. Importantly, the ClO_2_ released from the prepared smart sachets effectively preserved the quality of the cherry tomatoes for up to 16 d. Notably, fungal growth was reduced by >50% in commercial storage conditions. The versatility and efficacy of this newly developed ClO_2_-releasing system were demonstrated across three different scenarios, namely a closed system (simulating storage conditions), using clamshell packaging common to retail markets, and in corrugated boxes that are routinely used for mass shipment and wholesale distribution. It was found that this smart sachet offers an efficient method for preventing the microbial spoilage of fresh foods. However, further investigations are necessary to optimize its application for other food products using diverse packaging systems. This includes assessing the gas-release quantity in relation to the product quality and analyzing residual ClO_2_ levels. Future studies should also address critical factors such as the environmental impact, reactivity, and flammability of the residual ClO_2_. Moreover, evaluating the system performance across various food types with distinct characteristics is essential to evaluate the broader applicability of this system within the food industry.

## Figures and Tables

**Table 1:** Wavenumbers and assignments of the characteristic FT-IR bands recorded for CA, pure PEBAX, and the PEBAX/CA composite films.

Assignment	Characteristic Bands (cm^−1^)			
	CA	S_0_	S_1_	S_2_
O–H stretching	3292, 3495	845	845	845
C–O–C stretching	−	1100	1100	1100
C=O stretching	1699, 1742	1735	1731	1731
H–N–C=O stretching	−	1638	1638	1638
N–H stretching	−	3297	3297	3297
C–H bending	−	2856, 2948	2856, 2948	2856, 2948

**Table 2:** Thermal properties of the PEBAX/CA composite films.

Sample	DSC			TGA	
	T_g_ (°C) ^a^	T_m_ (°C) ^b^	ΔH_m_ (J/g) ^c^	T_1st_ (°C) ^d^	T_2nd_ (°C) ^e^
S_0_	−50.8 ± 0.2 ^f^	13.4 ± 0.6 ^f^	21.3 ± 0.9 ^f^	–	350–460
S_1_	−49.3 ± 0.3 ^g^	15.4 ± 0.4 ^g^	15.4 ± 1.5 ^g^	220–270	350–490
S_2_	−42.9 ± 0.5 ^h^	17.7 ± 0.4 ^h^	4.1 ± 0.8 ^h^	220–270	350–490

^a^ glass-transition temperature of the PEBAX phase in the PEBAX/CA composite film. ^b^ melting temperature of the PEBAX phase in the PEBAX/CA composite film. ^c^ melting enthalpy of the PEBAX phase in the PEBAX/CA composite film. ^d^ temperature range of the first thermal decomposition step of the PEBAX/CA composite film. ^e^ temperature range of the second thermal decomposition step of the PEBAX/CA composite film. Different letters within the same column indicate significant differences (*p* < 0.05).

**Table 3:** Results of ClO_2_ release test of sachets with different amounts of NaClO_2_. Two-way analysis of variance (ANOVA) showed significant effects of treatment (*p* < 0.001), storage time (*p* < 0.001), and their interaction (*p* < 0.001) on ClO₂ release.

Day	S_0_-0.1 g	S_1_-0.1 g	S_2_-0.1 g	S_0_-0.5 g	S_1_-0.5 g	S_2_-0.5 g	S_0_-1.0 g	S_1_-1.0 g	S_2_-1.0 g
0	0.00 ± 0.00 ^a^	0.00 ± 0.00 ^b^	0.00 ± 0.00 ^c^	0.00 ± 0.00 ^a^	0.00 ± 0.00 ^b^	0.00 ± 0.00 ^c^	0.00 ± 0.00 ^a^	0.00 ± 0.00 ^b^	0.00 ± 0.00 ^c^
1	0.00 ± 0.00 ^a^	5.75 ± 0.38 ^b^	0.61 ± 0.30 ^c^	0.00 ± 0.00 ^a^	4.69 ± 1.43 ^b^	5.98 ± 2.75 ^c^	0.00 ± 0.00 ^a^	3.83 ± 2.37 ^b^	5.71 ± 0.85 ^c^
2	0.00 ± 0.00 ^a^	8.37 ± 1.06 ^b^	7.51 ± 2.06 ^c^	0.00 ± 0.00 ^a^	6.73 ± 3.22 ^b^	6.08 ± 2.95 ^c^	0.00 ± 0.00 ^a^	15.48 ± 7.96 ^b^	14.96 ± 5.22 ^c^
3	0.00 ± 0.00 ^a^	7.31 ± 1.95 ^b^	13.01 ± 3.30 ^c^	0.00 ± 0.00 ^a^	13.93 ± 1.51 ^b^	6.34 ± 0.61 ^c^	0.00 ± 0.00 ^a^	17.16 ± 8.45 ^b^	16.16 ± 7.56 ^c^
4	0.00 ± 0.00 ^a^	8.87 ± 1.50 ^b^	16.02 ± 3.09 ^c^	0.00 ± 0.00 ^a^	18.53 ± 1.35 ^b^	8.25 ± 0.94 ^c^	0.00 ± 0.00 ^a^	19.04 ± 8.16 ^b^	18.29 ± 7.15 ^c^
5	0.00 ± 0.00 ^a^	9.62 ± 1.38 ^b^	17.06 ± 1.95 ^c^	0.00 ± 0.00 ^a^	22.15 ± 5.16 ^b^	8.41 ± 0.53 ^c^	0.00 ± 0.00 ^a^	19.29 ± 6.86 ^b^	21.63 ± 6.28 ^c^
6	0.00 ± 0.00 ^a^	10.02 ± 1.49 ^b^	17.40 ± 1.57 ^c^	0.00 ± 0.00 ^a^	24.51 ± 3.40 ^b^	8.86 ± 0.61 ^c^	0.00 ± 0.00 ^a^	22.53 ± 6.89 ^b^	23.29 ± 5.39 ^c^
7	0.00 ± 0.00 ^a^	10.48 ± 1.60 ^b^	17.91 ± 1.06 ^c^	0.00 ± 0.00 ^a^	27.61 ± 1.89 ^b^	9.45 ± 0.37 ^c^	0.00 ± 0.00 ^a^	21.32 ± 4.52 ^b^	24.82 ± 4.51 ^c^
8	0.00 ± 0.00 ^a^	10.88 ± 1.78 ^b^	18.40 ± 0.75 ^c^	0.00 ± 0.00 ^a^	28.45 ± 1.70 ^b^	9.91 ± 0.42 ^c^	0.00 ± 0.00 ^a^	19.11 ± 4.73 ^b^	26.14 ± 1.99 ^c^
9	0.00 ± 0.00 ^a^	11.27 ± 2.12 ^b^	18.61 ± 0.72 ^c^	0.00 ± 0.00 ^a^	28.16 ± 1.34 ^b^	10.33 ± 0.95 ^c^	0.00 ± 0.00 ^a^	20.94 ± 3.86 ^b^	27.16 ± 2.32 ^c^
10	0.00 ± 0.00 ^a^	11.45 ± 1.88 ^b^	18.81 ± 0.50 ^c^	0.00 ± 0.00 ^a^	28.20 ± 1.25 ^b^	10.59 ± 0.93 ^c^	0.00 ± 0.00 ^a^	19.87 ± 2.95 ^b^	27.16 ± 1.89 ^c^
12	0.00 ± 0.00 ^a^	11.68 ± 1.91 ^b^	18.81 ± 0.39 ^c^	0.00 ± 0.00 ^a^	27.76 ± 1.31 ^b^	10.88 ± 0.76 ^c^	0.00 ± 0.00 ^a^	19.64 ± 2.74 ^b^	26.88 ± 1.45 ^c^
15	0.00 ± 0.00 ^a^	14.11 ± 1.99 ^b^	19.32 ± 0.09 ^c^	0.00 ± 0.00 ^a^	27.61 ± 0.85 ^b^	12.61 ± 0.64 ^c^	0.00 ± 0.00 ^a^	19.15 ± 1.68 ^b^	27.48 ± 0.41 ^c^
18	0.00 ± 0.00 ^a^	15.76 ± 1.83 ^b^	19.79 ± 0.25 ^c^	0.00 ± 0.00 ^a^	27.05 ± 0.94 ^b^	13.15 ± 1.16 ^c^	0.00 ± 0.00 ^a^	18.93 ± 1.59 ^b^	27.50 ± 0.59 ^c^
21	0.00 ± 0.00 ^a^	16.03 ± 1.27 ^b^	19.59 ± 0.35 ^c^	0.00 ± 0.00 ^a^	27.56 ± 0.57 ^b^	15.64 ± 0.76 ^c^	0.00 ± 0.00 ^a^	19.92 ± 1.12 ^b^	28.27 ± 1.13 ^c^

Different letters indicate statistically significant differences within each column according to Duncan’s multiple range test (*p* < 0.05).

**Table 4:** Results of TSS, firmness, and pH of cherry tomatoes treated with sachets (S_0_, S_1_, and S_2_) during storage. Two-way ANOVA showed significant effects of treatment (*p* < 0.001), storage time (*p* < 0.001), and their interaction (*p* < 0.001) for all three parameters (TSS, firmness, and pH).

TSS			
Day	S_0_	S_1_	S_2_
0	7.57 ± 0.11 ^f^	7.57 ± 0.11 ^f^	7.57 ± 0.11 ^f^
3	7.05 ± 0.17 ^d^	7.30 ± 0.26 ^e^	7.23 ± 0.11 ^e^
6	6.90 ± 0.12 ^c^	7.10 ± 0.08 ^d^	7.15 ± 0.15 ^d^
9	6.78 ± 0.09 ^c^	7.07 ± 0.05 ^d^	7.22 ± 0.07 ^d^
12	6.48 ± 0.11 ^b^	6.72 ± 0.07 ^c^	6.72 ± 0.11 ^c^
16	6.27 ± 0.09 ^a^	6.57 ± 0.14 ^b^	6.55 ± 0.15 ^b^
Firmness			
Day	S_0_	S_1_	S_2_
0	8.29 ± 1.06 ^a^	8.29 ± 1.06 ^a^	8.29 ± 1.06 ^a^
3	7.98 ± 0.99 ^ab^	8.61 ± 1.14 ^b^	8.76 ± 1.01 ^b^
6	8.32 ± 1.11 ^b^	9.41 ± 0.79 ^c^	9.03 ± 1.17 ^bc^
9	8.64 ± 0.99 ^bc^	8.80 ± 0.91 ^bc^	8.92 ± 1.28 ^bc^
12	7.97 ± 1.16 ^ab^	8.87 ± 0.94 ^bc^	8.99 ± 1.04 ^c^
16	7.48 ± 1.28 ^a^	9.74 ± 1.22 ^c^	9.10 ± 1.24 ^c^
pH			
Day	S_0_	S_1_	S_2_
0	4.30 ± 0.02 ^a^	4.30 ± 0.02 ^a^	4.30 ± 0.02 ^a^
3	4.29 ± 0.02 ^a^	4.34 ± 0.02 ^b^	4.35 ± 0.03 ^b^
6	4.37 ± 0.02 ^b^	4.33 ± 0.02 ^b^	4.33 ± 0.02 ^b^
9	4.41 ± 0.01 ^c^	4.39 ± 0.01 ^c^	4.40 ± 0.01 ^c^
12	4.42 ± 0.03 ^cd^	4.40 ± 0.02 ^c^	4.44 ± 0.01 ^d^
16	4.67 ± 0.03 ^f^	4.52 ± 0.02 ^e^	4.54 ± 0.01 ^e^

Different letters indicate statistically significant differences within each column according to Duncan’s multiple range test (*p* < 0.05).

**Table 5:** Compositions of the PEBAX/CA composite film samples.

Sample Code [Series of PEBAX/CA]	Composition (*w*/*w*)	
	PEBAX	CA
S_0_ (Pure PEBAX)	100	0
S_1_	95	5
S_2_	90	10

## Data Availability

The original contributions presented in the study are included in the article, further inquiries can be directed to the corresponding author.

## Abbreviations

The following abbreviations are used in this manuscript:

CA	citric acid
ClO_2_	chlorine dioxide
FDA	Food and Drug Administration
FT-IR	Fourier transform infrared
NaClO_2_	sodium chlorite
PA	polyamide
PE	polyether
PEBAX	polyether-b-amide
PEO	polyethylene oxide
RH	relative humidity
SEM	scanning electron microscopy
TGA	thermogravimetric analysis
UTM	universal testing machine
